# Adherence to psychotropic medication in completed suicide in Sweden 2006–2013: a forensic-toxicological matched case-control study

**DOI:** 10.1007/s00228-019-02707-z

**Published:** 2019-06-19

**Authors:** Jonas Forsman, Heidi Taipale, Thomas Masterman, Jari Tiihonen, Antti Tanskanen

**Affiliations:** 1 Department of Clinical Neuroscience, Karolinska Institutet, Stockholm, Sweden; 2 National Board of Forensic Medicine, PO Box 4044, SE-141 04 Huddinge, Sweden; 3 School of Pharmacy, University of Eastern Finland, Kuopio, Finland; 4 Department of Forensic Psychiatry, Niuvanniemi Hospital, Kuopio, Finland

**Keywords:** Pharmacoepidemiology, Antidepressives, Antipsychotics, Compliance, Suicide

## Abstract

**Objective:**

To investigate the influence of adherence to psychotropic medications upon the risk of completed suicide by comparing person-level prescriptions and postmortem toxicological findings among complete-suicide cases and non-suicide controls in Sweden 2006–2013.

**Methods:**

Using national registries with full coverage on dispensed prescriptions, results of medico-legal autopsies, causes of death, and diagnoses from inpatient care, estimated continuous drug use for 30 commonly prescribed psychotropic medications was compared with forensic-toxicological findings. Subjects who had died by suicide (cases) were matched (1:2) with subjects who had died of other causes (controls) for age, sex, and year of death. Odds ratios were calculated using logistic regression to estimate the risk of completed suicide conferred by partial adherence and non-adherence to pharmacotherapy. Adjustments were made for previous inpatient care and the ratio of initiated and discontinued dispensed prescriptions, a measure of the continued need of treatment preceding death.

**Results:**

In 5294 suicide cases and 9879 non-suicide controls, after adjusting for the dispensation ratio and other covariates, partial adherence and non-adherence to antipsychotics were associated with 6.7-fold and 12.4-fold risks of completed suicide, respectively, whereas corresponding risk estimates for antidepressant treatment were not statistically significant and corresponding risk increases for incomplete adherence to antidepressant treatment were lower (1.6-fold and 1.5-fold, respectively) and lacked statistical significance.

**Conclusion:**

After adjustment for the need of treatment, biochemically verified incomplete adherence to antipsychotic pharmacotherapy was associated with markedly increased risks of completed suicide.

**Electronic supplementary material:**

The online version of this article (10.1007/s00228-019-02707-z) contains supplementary material, which is available to authorized users.

## Introduction

Associations between the use of psychotropic medications and the risk of suicidality are a topic of recurrent discussion. Whereas antipsychotic agents—particularly clozapine and the mood-stabilizer lithium—seem to have protective effects on both non-lethal suicidality and completed acts of suicide in schizophrenia and affective disorders [1–4], concerns have been raised regarding antidepressants [5–9]. Indeed, despite numerous large, population-based studies investigating possible antisuicidal or prosuicidal properties of antidepressants, results regarding their overall effect on suicide risk remain inconclusive [10–13].

In the few studies that have demonstrated protective effects for psychotropic agents on the suicide risk, the effects appear to be more specifically associated with continuous medication use [3, 14–18]. Although poor adherence to, discontinuation of, and switches between psychotropic medications have been identified as reproducible risk factors for suicide, early discontinuation of antidepressant treatment has been reported to decrease suicide risk in the elderly [19, 20]. Further, in two studies matching for suicidal propensity in Canada and Sweden, initiation of selective-serotonin-reuptake-inhibitor therapy increased the risk of violent suicide during the first month of treatment [21, 22].

In a few studies of completed suicide, adherence to psychotropic medications has been confirmed by biochemical methods, but never in comparison to adherence in non-suicide controls [23–25]. Further, even in registry-based studies including controls, discrepancies exist regarding how continuous medication use has been operationalized vis-à-vis the timing of prescription dispensation [26, 27]. Also, whereas non-biochemical registry-based studies rely on the assumption that single or consecutive medication purchases reflect genuine intake, toxicological analyses can verify medication use in relation to therapeutic concentrations.

Apart from published reports using Swedish registry data to assess adherence to non-addictive medications in the general population [28]; to psychotropic medications in homicide offenders and victims [29]; and to antidepressants among young suicide victims [30], no previous study has exploited discrepancies between person-level prescription and toxicology data derived from nationwide registries to investigate biochemically verified pharmacoadherence.

### Aims of the study

In this population-based forensic-toxicological study with a matched case-control design, our primary objective was to investigate biochemically verified adherence to treatment with psychotropic medications in all instances of completed suicide (by means other than self-poisoning; cases) and instances of non-suicide deaths (controls) occurring in Sweden 2006–2013. A second objective was to investigate a novel measure of the continued need for treatment based on the ratio of initiated and discontinue prescriptions. Based upon previous research implying that pharmacoadherence to psychotropic agents reduces suicide risk, we hypothesized that toxicologically verifiable incomplete adherence to antidepressant and antipsychotic medications would be associated with increased risks of completed suicide.

## Material and methods

### Setting

During forensic autopsies—conducted by the National Board of Forensic Medicine (NBFM) to clarify the cause of death in instances of suspected unnatural death—blood samples are routinely collected for toxicological analyses performed using a test panel capable of detecting the most common legal and illegal pharmacologically active substances and their metabolites [31]. Results of toxicological analyses and causes of death are registered, respectively, in the NBFM database Toxbase and the National Board of Health and Welfare’s (NBHW) Cause of Death Registry. NBHW’s Prescription Registry contains complete individual-level information for all dispensed prescriptions since July 1, 2005, while its National Patient Registry contains individual-level information for all instances of inpatient care since 1987. For this study, we linked complete records, retrieved from Toxbase, for all forensically investigated deaths in Sweden during the period January 1, 2006, to December 31, 2013, to NBHW’s prescription and patient registries. For further comparisons between study subjects and the Swedish general population, official annual statistics were retrieved from publicly available statistical databases at Statistics Sweden and NBHW.

### Subjects

**Fig. 1:**
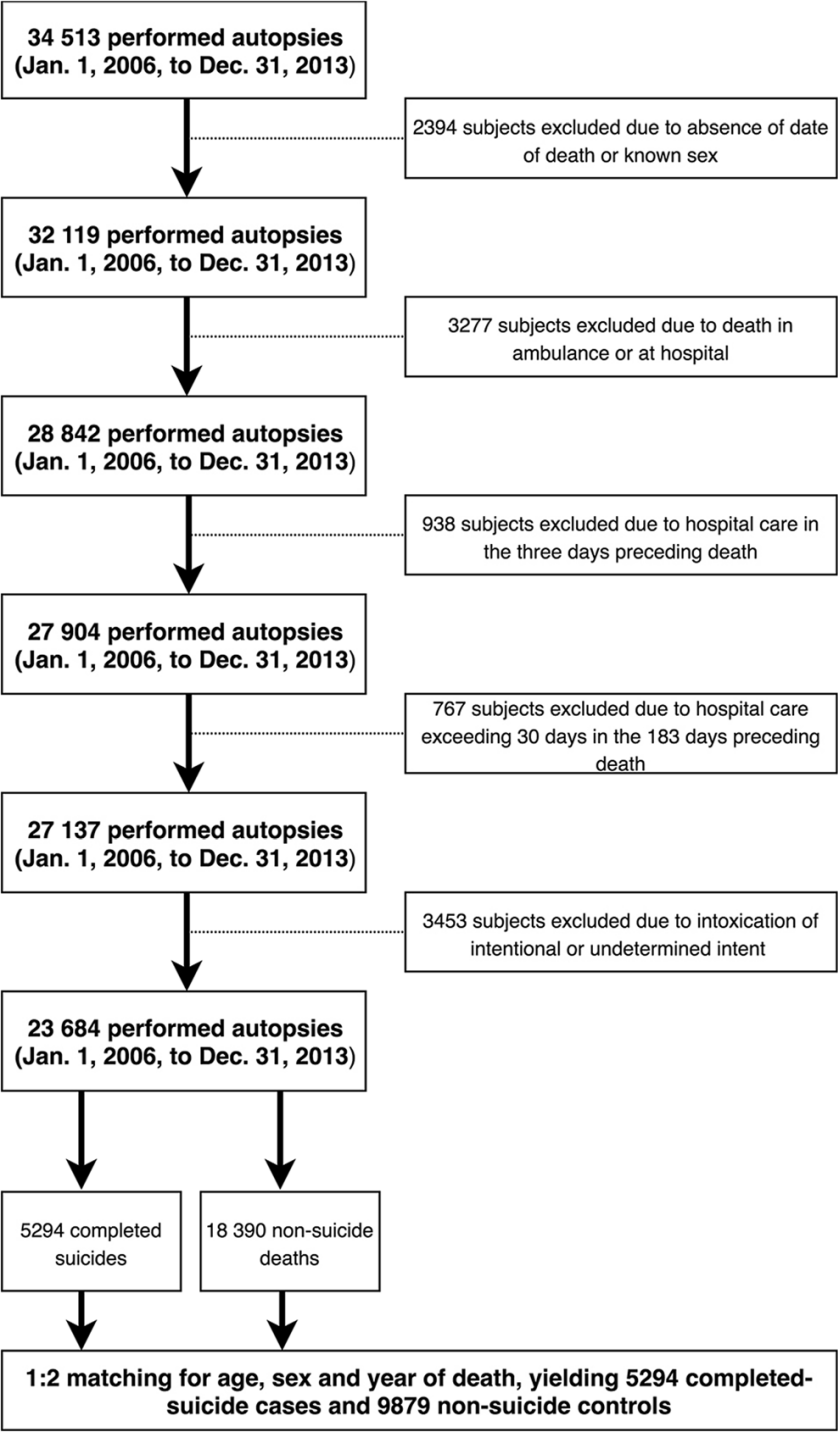
Flow chart of exclusions for the study sample

From 34,513 forensic autopsies, complete records regarding age, sex, date and cause of death, postmortem toxicological findings, and prescription history were available for 23,684 subjects, after exclusion of 2394 subjects for whom information on date of death or sex was unavailable; 4982 subjects who had died in an ambulance or at hospital, been admitted to a hospital in the 3 days preceding death, or spent more than 30 days cumulatively on a hospital ward during the 183 days preceding death (to remove instances of non-registered pharmacological treatment from ambulance or hospital care); and 3453 subjects who had died from self-poisoning of intentional or undetermined intent (ICD-9 codes E966-E969; and ICD-10 codes X60-X69 and Y10-Y19; to minimize the possibility of false-positive pharmacoadherence; Fig. 1). Approximate 1:2 matching was performed for age, sex, and year of death, rendering 5294 completed-suicide cases and 9879 non-suicide controls. Prevalence of comorbid conditions from previous inpatient care was operationalized as one possible main diagnosis per person and year. The study was approved by the Regional Ethical Review Board in Stockholm (2013/1411-31/5).

### Analyses

Virtually all deaths investigated by NBFM undergo initial screening for nearly 200 substances by the use of liquid-chromatography time-of-flight technology. Upon positive finding, substances’ concentrations are then quantified by gas chromatography–mass spectrometry or liquid-chromatography–tandem mass spectrometry and recorded in Toxbase [32]. The methods have been accredited with validated detection thresholds for analyzed substances.

### Exposure

All psychotropic medications whose active substances or metabolites NBFM routinely screens for were included, with the exception of addictive substances readily acquired illegally (benzodiazepines and stimulants). To minimize the risk of underestimation of adherence—which, in the presence of differences in treatment regimens between cases and controls, could give rise to spurious associations—included substances were required to have half-lives longer than 5 h and to be detectable at concentrations near the lower limit of the recognized therapeutic range (Supplementary table 3) [33]. Possible variations in substances’ postmortem blood dilution were accounted for as in previous studies [34–37]. Altogether, data on 30 psychotropic medications were modeled and compared with corresponding forensic-toxicological findings. All medications were categorized according to the Anatomical Therapeutic Chemical classification system and, in accordance with intended indications in a psychiatric setting, defined as antidepressants (N06A) or antipsychotics (N05A, excluding lithium).

### PRE2DUP

Expected medication-use periods were modeled using the Prescription Registry by PRE2DUP, as previously described [26, 28]. The method is based on sliding averages of daily defined doses (DDD) calculated for each medication over time, taking personal medication-purchasing behavior and medication stockpiling into account; in the case of single purchases, the expected duration of the purchased package was used [38]. In this study, the last purchase was the primary evaluated factor when medication use at the time of death was assessed; dates of death were not known to PRE2DUP modelers (HT, AT). A prescription history of at least 365 days before death was available for all included subjects, except for subjects who had died before July 1, 2006.

### Statistics

Based on modeled medication data, total purchased DDDs, total number of changes (initiations and discontinuations) of dispensed prescriptions, and dispensation ratio (the number of initiations divided by the number of discontinuations) with 95% confidence intervals (CIs) were calculated for the year preceding death (or the 183 days preceding death for subjects who had died before July 1, 2006) for: psychotropic medications whose active substances or metabolites met toxicological inclusion criteria; all dispensed psychotropic medications; and all dispensed medications. Above calculations were also performed separately for antidepressants and antipsychotics. Dispensation ratios equal or exceeding 1.0 were interpreted as representing ongoing monotherapy or increasing polypharmacy and thus conceptualized as a measure of continued need of treatment.

Assessment of agreement between PRE2DUP-modeled medication use and toxicological findings as a measure of biochemically verified pharmacoadherence was made at an individual level for all toxicologically investigated psychotropic medications. Risks of completed suicide conferred by adherent, partially adherent or non-adherent use of each class of psychotropic medication, were estimated using logistic regression by comparing exposure to treatment in cases and controls expressed as odds ratios (ORs) with 95% CIs. ORs for which uncorrected 95% CIs did not span unity were considered statistically significant.

### Criteria of judgment

Adherence was defined as congruence between the predicted occurrence of continuous medication use at the time of death and positive postmortem toxicology for the same medication’s active substance or metabolite, without simultaneous incongruence for a medication in the same class. Non-adherence was defined as predicted occurrence of continuous medication use at the time of death in the absence of positive postmortem toxicology for all medications in a particular class, and partial adherence as simultaneous congruence and incongruence for multiple medications in the same class.

Adjustments were made for sex, age, previous inpatient care, and previous psychiatric inpatient care (both defined as at least one instance of inpatient care recorded in the National Patient Registry), as well as the dispensation ratio during the 4 months preceding death. Statistical analyses were performed using R version 3.4 .4 (R Core Team, Vienna, Austria) and SPSS version 22.0 (IBM Corp., Armonk, NY, USA).

### Ethics

Our investigation was carried out in accordance with the latest version of the Declaration of Helsinki. The research ethics committee in Stockholm, Sweden, approved the current research (reference number 2013/1411-31/5).

### Data availability

The data that support the findings of this study are available from the two Swedish governmental agencies: the Swedish National Board of Forensic Medicine’s department of forensic toxicology (Rättsmedicinalverket - Rättskemi; Toxbase; http://www.rmv.se); and the National Board of Health and Welfare (Socialstyrelsen; Prescription Registry and Cause of Death Registry; http://www.sos.se), but restrictions apply to the availability of these data, which were used under license for the current study, and so are not publicly available. Data are however available from the authors upon reasonable request and with permission from Rättsmedicinalverket and Socialstyrelsen.

## Results

### Characteristics of sample

As seen in Table 1, of 5294 completed-suicide cases, 80% were male and 73% under 65 years of age (mean age 52 years, SD 19.9 years), whereas, of 9879 non-suicide controls, 82% were male and 72% under 65 years of age (mean age 53 years, SD 19.1). In comparison, for the study period, the living Swedish population consisted of nearly equal distribution of men and women, with a mean age of 41 years, with 82% under 65 years of age. After exclusion of self-poisoning, the two most common main causes of death among controls were death due to external causes (ICD-10 codes V01-Y98, with the exception of Y10-Y19), at 39%, and death resulting from diseases of the circulatory system (ICD-10 codes I00-I99), at 37%. In the Swedish population, for the study period, main causes of death were death resulting from diseases of the circulatory system, at 40%, and neoplasms (ICD-10 codes C00-D48), at 25%, whereas the average annual proportion of individuals dying from external causes was 5%. The contribution of each of the 8 years of the study period to the total number of cases and controls was relatively constant.

**Table 1:** Characteristics of the sample included in the study after matching by age, sex, and year of death

Variable	Completed-suicide cases	Non-suicide controls	Swedish general population^†^
	*N* = 5294	*N* = 9879	*N* = 9,374,049
Age			
Mean in years (SD)	51.5 (19.9)	53.1 (19.1)	41.1
Proportion under 65 years of age (*N*)	73.04% (3867)	71.67% (7080)	81.67% (7,655,535)
Proportion over 65 years of age (*N*)	26.96% (1427)	28.33% (2799)	18.33% (1,718,514)
Proportion females (*N*)	20.06% (1062)	19.18% (1895)	50.2% (4,706,861)
Proportions of the main causes of death^‡^ (*N*)			
C00-D48 (neoplasms)	0.04% (2)	1.30% (122)	25.00% (22,746)
E00-E90 (endocrine, nutritional, and metabolic diseases)	0.09% (5)	2.74% (271)	2.65% (2412)
F00-F99 (mental and behavioral disorders)	0.08% (4)	5.23% (517)	5.76% (5242)
G00-G99 (diseases of the nervous system)	0.02% (1)	1.98% (196)	3.81% (3476)
I00-I99 (diseases of the circulatory system)	0.83% (44)	37.18% (3673)	39.79% (36,199)
J00-J99 (diseases of the respiratory system)	0.06% (3)	4.40% (435)	6.32% (5746)
K00-K93 (diseases of the digestive system)	0.19% (10)	5.39% (532)	3.16% (2875)
R00-R99 (symptoms, signs, and abnormal clinical and laboratory findings, not elsewhere classified)	0.51% (27)	2.45% (242)	3.57% (324)
V01-Y98 (external causes of morbidity and mortality)	98.19% (5198)	38.69% (3822)	5.19% (4721)
Proportions dying each year (*N*)			
2006	13.37% (708)	13.09% (1293)	12.54% (91,270)
2007	12.45% (659)	12.66% (1251)	12.62% (91,815)
2008	13.01% (689)	12.72% (1257)	12.58% (91,541)
2009	12.94% (685)	12.80% (1265)	12.39% (90,172)
2010	12.84% (680)	12.99% (1283)	12.44% (90,518)
2011	11.41% (604)	11.19% (1105)	12.36% (89,941)
2012	11.47% (607)	11.93% (1179)	12.64% (92,020)
2013	12.50% (662)	12.61% (1246)	12.44% (90,527)
Proportion positive for alcohol (*N*)	25.88% (1370)	25.78% (2547)	NA
Proportion positive for other GABAergic hypnotics (*N*)	18.32% (970)	17.92% (1770)	NA
Proportion positive for opioids (*N*)	3.04% (161)	7.81% (772)	NA
Proportion positive for other narcotics (*N*)	2.83% (150)	5.92% (585)	NA
Proportions of comorbid conditions contributing to cause of death^§^ (*N*)			
Diabetes mellitus	0.21% (11)	4.19% (414)	NA
Heart failure	0.23% (12)	3.25% (321)	NA
Ischemic heart disease	2.02% (107)	30.53% (3016)	NA
Hypertension	0.02% (1)	0.35% (35)	NA
Asthma/chronic obstructive pulmonary disease	0.17% (9)	1.79% (177)	NA
Psychosis	0.09% (5)	0.11% (11)	NA
Bipolar disorder	0.09% (5)	0.00% (0)	NA
Alcohol-related disorders	6.54% (346)	20.44% (2019)	NA
Drug-related disorders	0.30% (16)	2.15% (212)	NA
Proportion of comorbid conditions (main diagnosis) in inpatient care during study period^§^ (*N*)			
Diabetes mellitus	0.8% (43)	3.1% (307)	1.2% (109,848)
Heart failure	1.4% (74)	2.3% (230)	2.9% (261,170)
Ischemic heart disease	2.6% (136)	2.8% (281)	5.5% (502,559)
Hypertension	0.4% (21)	0.5% (50)	3.1% (280,072)
Asthma/chronic obstructive pulmonary disease	0.9% (47)	2.0% (201)	2.1% (186,810)
Psychosis	4.8% (256)	3.0% (294)	1.2% (109,271)
Bipolar disorder	2.6% (139)	0.9% (91)	0.6% (49,807)
Depression	10.6% (560)	2.1% (211)	1.2% (108,207)
Alcohol-related disorders	10.6% (563)	23.8% (2349)	3.3% (296,142)
Drug-related disorders	3.6% (188)	7.6% (750)	0.6% (51,404)

^†^Mean values for living and deceased individuals in the Swedish general population during the study period, 2006–2013

^‡^For complete list of main causes of death, see Supplementary Table 1

^§^For complete list of included ICD-10 diagnoses, see Supplementary Table 2

Rates of positivity for ethanol and GABAergic hypnotics upon toxicological analysis were, after rounding, equal in completed-suicide cases and non-suicide controls, at 26% and 18%, respectively, whereas opioids and other addictive medications were less commonly detected in cases than in controls (3% vs. 8%, and 3% vs. 6%, respectively). Alcohol-related disorders and ischemic heart disease were the two most common comorbid conditions adjudged to have contributed to the cause of death in both cases (at 7% and 2%, respectively) and controls (at 20% and 31%, respectively). Comorbid conditions contributing to the main causes of death were not available for the general population. Among completed-suicide cases, depression (10.6%), alcohol-related disorders (10.6%), and psychosis (4.8%) were the most common main diagnoses from previous inpatient care, whereas in non-suicide controls, alcohol-related disorders (23.8%), drug-related disorders (7.6%), and diabetes mellitus (3.1%) were the leading main diagnoses. For all main diagnoses other than pulmonary or cardiovascular conditions, the general population showed a lower prevalence of previous inpatient care compared with the study group (Table 1).

### Dispensation ratios

The number of changes in dispensed prescriptions per person, the dispensation ratio per month, and DDD per person were modeled for the year preceding death in cases and controls for all medications, all psychotropic medications, and all toxicologically verifiable psychotropic medications. Among cases, the monthly number of changes per person increased over the year preceding death (all medications 0.56–1.10; all psychotropic medications 0.18–0.48; and verifiable psychotropic medications 0.05–0.15), while non-suicide controls showed similar trends (all medications 1.03–1.49; all psychotropic medications, 0.27–0.39; and verifiable psychotropic medications 0.05–0.08). Increases were more dependent on initiated prescriptions in cases than in controls, as shown by differences between groups regarding dispensation ratios. Calculated linear fitting for the 6 months preceding death showed statistically significant slopes of similar magnitude for changes in dispensation ratios, but in opposite directions, between completed-suicide cases (positive slope) and non-suicide controls (negative slope), for all psychotropic medications and toxicologically verifiable psychotropic medications. By the last month prior to death, cases had surpassed controls with regard to dispensed DDDs per person per month for all psychotropic medications and verifiable psychotropic medications (Supplementary table 4).

**Fig. 2:**
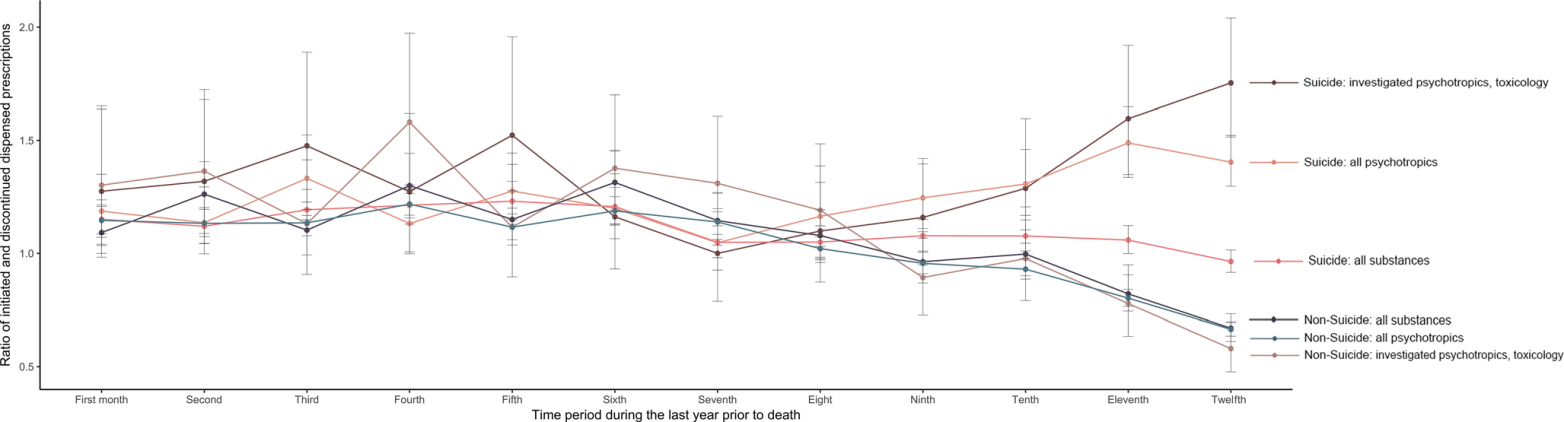
Ratio of initiated and discontinued dispensed prescriptions during the last year prior to death among suicide and non-suicide deaths

In completed-suicide cases, the dispensation ratio decreased for all medications (1.15–0.96) yet increased for all psychotropic medications (1.17–1.42) and verifiable psychotropic medications (1.30–1.76), in the year preceding death, whereas ratios in non-suicide controls decreased for all types of prescriptions (all medications, 1.15–0.66; all psychotropic medications, 1.11–0.67; and verifiable psychotropic medications, 1.29–0.59). By 2 months prior to death, 95% CIs for dispensation ratios of cases and controls no longer overlapped for any prescription type (Supplementary table 4, Fig. 2).

Differences in dispensation ratios for verifiable psychotropic substances were further modeled with a focus on antidepressants and antipsychotics. The ratio in completed-suicide cases prescribed antidepressants increased in the months prior to death, diverging, at 2 months prior to death, from ratios in other groups; by the last month prior to death, the 95% CI for the ratio in cases no longer overlapped with intervals for ratios in other groups (Supplementary figure 1, Supplementary table 5).

### Adherence and suicide risk

In models comparing risk estimates for completed suicide conferred by degrees of adherence to antipsychotics and antidepressants, adherence was set as reference (OR 1; Table 2). Unadjusted risk estimates for non-adherence and partial adherence to antipsychotics were OR 9.51 (95% CI 2.70–60.30) and OR 4.59 (95% CI 1.30–29.16), respectively. Following adjustments for psychiatric and somatic inpatient care, non-adherence and partial adherence conferred, respectively, the highest and next highest risk estimates (adjusted OR [aOR] 9.92, 95% CI 2.80–63.14 and aOR 4.72, 95% CI 1.33–30.10). After further adjustment for dispensation ratio, risk estimates increased further for both non-adherence (aOR 12.43, 95% CI 2.06–238.66) and partial adherence (aOR 6.66, 95% CI 1.20–128.04). For antidepressants, unadjusted risk estimates for non-adherence and partial adherence were OR 1.61 (95% CI 1.05–2.50) and OR 1.57 (95% CI 1.04–2.40), respectively. Adjustments for previous psychiatric and somatic inpatient care yielded attenuated risk estimates for suicide, with the risked conferred non-adherence being statistically significant (partial adherence, aOR 1.43, 95% CI 0.94–2.20; non-adherence, aOR 1.59, 95% CI 1.03–2.49); however, after a final adjustment for dispensation ratio, the latter risk estimate was no longer significant (aOR 1.52, 95% CI 0.67–3.44). Analyses of possible interactions between sex and adherence status revealed no significant effects for any combination (data not shown).

**Table 2:** Odds ratios with 95% confidence intervals for completed suicide conferred by biochemically verified partial adherence and non-adherence (defined, respectively, as partial congruence and incongruence between the prescription-based measure of continuous medication use and postmortem forensic-toxicological findings)

		Completed-suicides:non-suicide deaths	OR (95% CI)	aOR (95% CI)^†^	aOR (95% CI)^‡^
Antipsychotics	Adherence	2:18	1.0 (reference)	1.0 (reference)	1.0 (reference)
	Partial adherence	128:251	4.59 (1.30–29.16)	4.72 (1.33–30.10)	6.66 (1.10–128.04)
	Non-adherence	207:196	9.51 (2.70–60.30)	9.92 (2.80–63.14)	12.43 (2.06–238.66)
Antidepressants	Adherence	39:55	1.0 (reference)	1.0 (reference)	1.0 (reference)
	Partial adherence	1186:1064	1.57 (1.04–2.40)	1.43 (0.94–2.20)	1.58 (0.71–3.53)
	Non-adherence	415:363	1.61 (1.05–2.50)	1.59 (1.03–2.49)	1.52 (0.67–3.44)

^†^Adjusted for age, sex, previous psychiatric inpatient care, and previous somatic inpatient care

^‡^Adjusted for ^†^ and dispensation ratio during the 4 months preceding death

*CI* confidence interval, *OR* odds ratio, *aOR* adjusted odds ratio

## Discussion

This nationwide postmortem study included information regarding nearly all incidents of suspected unnatural death in Sweden 2006–2013. Using a matched case-control design, we investigated whether biochemically verified incomplete adherence to commonly prescribed psychotropic medications influences the risk of completed suicide. To our knowledge, this is the first time that the combined methods of pharmacoepidemiology and toxicology have been complemented with data concerning non-suicide deaths to address, by means of a case-control design, the influence of pharmacoadherence on suicide risk. Moreover, this is the first population-based study in which the ratio of initiated and discontinued prescriptions for psychotropic medications has been operationalized as a proxy for continued need of treatment.

### Pharmacoadherence and completed suicide

The main result of the present study was that, in comparison with adherent use, both partial adherence and non-adherence to treatment with antidepressant and antipsychotic medications confer increased risks of completed suicide. However, after adjustments for variables reflecting the degree of somatic and psychiatric illness and continued need of psychotropic treatment, risk estimates for antidepressants were no longer significant. For antipsychotics, the latter adjustment resulted in risk-estimate increases for both partial adherence (from 4.72 to 6.66) and non-adherence (from 9.92 to 12.43), suggesting that a higher degree of continued treatment need was associated with a greater level of adherence (perhaps reflecting the use of long-acting injectables in severe psychosis).

An immediately evident explanation for the finding that partial adherence and non-adherence to psychotropic medication confer increased suicide risks is that discontinuation of a prescribed medication results in cessation of its pharmacodynamic antidepressive or antipsychotic effects, since states of depression and psychosis are well-known risk factors for suicide [39, 40]. Given the design of the study, however, it is difficult to draw convincing conclusions regarding causality. Indeed, another possibility is that the degree of mental illness is inversely related to adherence—that individuals with a greater burden of illness are less capable of sustaining continuous medication treatment, affecting the course of their illness [41]. A third possibility is that switching or discontinuation of psychotropic medications may give rise to withdrawal or rebound phenomena, whereby symptoms for which medications were originally prescribed, including suicidality, reoccur [42, 43].

### Dispensations ratio

A subsidiary result of the study was that the average dispensation ratios in completed-suicide cases and non-suicide controls diverged markedly in the last month prior to death. In a further comparison of prescriptions for toxicologically verifiable psychotropic medications, the ratio for dispensed antidepressants in cases (2.1) exceeded the ratio for dispensed antidepressants in controls (0.6), as well as the ratios for dispensed antipsychotics in both cases (0.9) and controls (0.7). Since ratios exceeding 1.0 represent an increase in prescribed medications (newly initiated use of a single medication, or initiation of additional medications in the case of concomitant use), they are assumed to indicate the need of augmented treatment, for reasons that may include insufficient treatment response and increased severity of symptoms and signs, including suicidality. As the number of changes increased at a greater rate than the simultaneous increase in the dispensation ratio, in addition to the initiation of medications and restarting of previous non-continuous drug use, switching must also have occurred.

Although switching medications drives the dispensation ratio towards 1.0, it may nonetheless indicate a need for augmented treatment. In fact, recent findings suggest that switching of antidepressants confers a more than the two-fold elevated risk of suicidality in late life [44], yet, in the present study, adjustments for dispensation ratio had little effect on risk estimates for incomplete adherence to antidepressants. By contrast, the same adjustment increased risk estimates for incomplete adherence to antipsychotics, reflecting a difference between cases and controls regarding patterns of use of psychotropic medications in the months prior to death. Thus, it may be the case that non-suicidal individuals are less often treated with psychotropic medications, or that changes of psychotropic treatment entail the combined disadvantages of initiation side effects, discontinuation syndromes, and treatment gaps.

### Strengths and limitations

The present dataset comprises reliable forensic-toxicological findings from a virtually complete national record of incidents of suspected unnatural death linked to individual-level information on all dispensed prescriptions. Further, by excluding substances with short half-lives, we compensated for lack of information regarding exact times of death and last administrated doses. One limitation is related to the possibility of intermittent use of prescribed medications. The calculated rates of adherence may therefore be overestimations of “long-term adherence,” but, at the same time accurate measures of “short-term adherence”—a phenomenon which, nonetheless, in itself, might influence suicide risk. Exclusion of deaths by intoxication should, in this study, be seen as both a strength and a limitation. Upon forensic investigation, an overdose of prescribed medications confounds judgments regarding pharmacoadherence, and level of suicidal intentionality is more difficult to determine in medication intoxications [45]. Yet, exclusion of intoxications meant that nearly a third of all instances of completed suicide in the study period were unaccounted for—and younger individuals and women underrepresented—limiting the generalizability of the results. Owing to the phenomena of postmortem redistribution—whereby concentrations of substances in the blood generally get elevated—it is conceivable that we have misclassified non-adherence subjects as adherent. Finally, although all investigated categories of pharmacoadherence presuppose a medical indication for treatment, lack of reliable clinical outpatient data precluded more precise adjustment for possible residual confounding by indication.

### Clinical implications

Controls in our dataset showed a greater burden of somatic and psychiatric illness—recognized risk factors for both incomplete adherence and suicide—than in the general population. Thus, reported risk estimates for completed suicide are likely conservative underestimations, implying even superior real-world effectiveness regarding suicide risk for wholly adherent use of psychotropic medications. The most common first line of pharmacological treatment for (suicidal) depressive states is antidepressants. Although prior research has demonstrated antidepressive effects of augmentation with antipsychotics in isolated major depressive disorder, antipsychotics are rarely prescribed in the absence of co-occurring signs of psychosis or mania [46, 47]. Based on our results, we suspect that individuals prone to violent suicidality might benefit from adherent use of antipsychotics. However, owing to the lack of information regarding specific indications for investigated psychotropic medications, we cannot with certainty conclude that antipsychotics exert a general antisuicidal effect. Still, we recommend that antipsychotics be considered for the treatment of suicidality when other forms of treatment have failed—all the more since poor adherence to antipsychotics can be countered by the use of long-acting injectables. Finally, our results support the notion that in suicidal individuals treated with psychotropic medications, especially antidepressants, warning signs for adverse outcomes may be low adherence, switching of medications, and markedly increased polypharmacy. Upon the emergence of such signs, the threshold for inpatient care should be lowered to prevent suicidal behavior.

## Electronic supplementary material

ESM 1 (PDF 2128 kb)

ESM 2 (PDF 1015 kb)
