# Correction to: Adherence to psychotropic medication in completed suicide in Sweden 2006–2013: a forensic-toxicological matched case-control study

**DOI:** 10.1007/s00228-026-03994-z

**Published:** 2026-01-28

**Authors:** Jonas Forsman, Heidi Taipale, Thomas Masterman, Jari Tiihonen, Antti Tanskanen

**Affiliations:** 1 Department of Clinical Neuroscience, Karolinska Institutet, Stockholm, Sweden; 2 National Board of Forensic Medicine, PO Box 4044, SE-141 04 Huddinge, Sweden; 3 School of Pharmacy, University of Eastern Finland, Kuopio, Finland; 4 Department of Forensic Psychiatry, Niuvanniemi Hospital, Kuopio, Finland

**Correction to : European Journal of Clinical Pharmacology (2019) 75:1421–1430**

10.1007/s00228-019-02707-z

Following publication, the authors were made aware that the toxicological analytical methods for certain included substances were not applied consistently across the entire study period. Specifically, variations in detection limits and related analytical criteria were identified between time periods, resulting in non-uniform measurement conditions for a subset of observations. In response, we conducted a comprehensive re-evaluation of the dataset and excluded all observations originating from time periods in which the predefined detection limits and analytical quality criteria were not met. This correction yielded updated numerical values in the affected analyses.

## Impact on Conclusions

All corrections are **numerical** and do **not affect the study’s results, interpretation, or conclusions**.

The authors apologize for these errors.

The following corrections has been made to Table 2.

Table 2 Odds ratios with 95% confidence intervals for completed suicide conferred by biochemically verified partial adherence and non-adherence (defined, respectively, as partial congruence and incongruence between prescription-based measure of continuous medication use and postmortem forensic-toxicological findings)

**Table:** 

		**Completed suicides: non-suicide deaths**	**OR (95% CI)**	**aOR (95% CI) **^**†**^	**aOR (95% CI)**^**‡**^
**Antipsychotics**	Adherence	2:18	1·0 (reference)	1·0 (reference)	1·0 (reference)
	Partial Adherence	**124:249**	**4·73 (1·41-31·89)**	**5·01 (1·41-31·89)**	**8·67 (1·44-166·92)**
	Non-Adherence	**112:122**	**9·97 (2·78-63·83)**	**9·97 (2·78-63·83)**	**13·19 (2·17-255·12)**
**Antidepressants**	Adherence	**18:39**	1·0 (reference)	1·0 (reference)	1·0 (reference)
	Partial Adherence	**1175:1072**	**2·19 (1·23-4·03)**	**1·93 (1·07-3.58)**	**1·86 (0·69-5·22)**
	Non-Adherence	**356:307**	**2·32 (1·29-4·33)**	**2·22 (1·22-4·18)**	**1·82 (0·66-5·19)**

† adjusted for age, sex, previous psychiatric inpatient care, and previous somatic inpatient care

‡ adjusted for † and dispensation ratio during the four months preceding death

Abbreviations: CI = confidence interval, OR = odds ratio, aOR = adjusted odds ratio

The following corrections has been made to Supplementary table 3.

Supplementary table 3. Elimination half-lives, detection thresholds, therapeutic ranges, and postmortem redistribution factors for the studied drugs

**Table:** 

**ATC**^**1**^	**Substance**	**ATC class**^**1**^	**Minimum elimination half-life (h) **^**2**^	**Detection**^**threshold**^(mg/L) ^* 3^	**Therapeutic range (mg/L)**^**4**^	**Postmortal redistribution factor**^**5-7**^
N05AA01	Chlorpromazine	Antipsychotic	30·0	0·10	0·03-0·10	4·0
N05AB01	Dixyrazine	Antipsychotic	5·0	0·20	0·30--	
N05AC02	Thioridazine	Antipsychotic	21·0	0·20	0·20-2·00	1·2
N05AD01	Haloperidol **	Antipsychotic	12·0	0·01	0·005-0·017	3·6
N05AE04	Ziprasidone **	Antipsychotic	6·5	0·01	0·05-0·20	
N05AF05	Zuclopenthixol **	Antipsychotic	20·0	0·01	0·004-0·05	
N05AH02	Clozapine	Antipsychotic	6·0	0·05	0·10-0·60	2·8
N05AH03	Olanzapine	Antipsychotic	30·0	0·02	0·02-0·08	2·7-23·0
N05AH04	Quetiapine	Antipsychotic	7·0	0·02	0·10-0·50	9·0
N05AX08	Risperidone **	Antipsychotic	23·0	0·01	0·006-0·02	
N05AX12	Aripiprazole **	Antipsychotic	25·0	0·02	0·15-0·50	0·4
N05AX13	Paliperidone **	Antipsychotic	23·0	0·01	0·02-0·06	
N06AA04	Clomipramine	Antidepressant	21·0	0·05	0·02-0·40	1·9
N06AA09	Amitriptyline	Antidepressant	25·0	0·05	0·05-0·30	
N06AA10	Nortriptyline	Antidepressant	18·0	0·10	0·02-0·20	
N06AA21	Maprotiline	Antidepressant	27·0	0·10	0·075-0·130	3·4
N06AC01	Maprotiline	Antidepressant	27·0	0·10	0·075-0·130	3·4
N06AB03	Fluoxetine	Antidepressant	96·0	0·02	0·12-0·50	1·6-2·9
N06AB04	Citalopram	Antidepressant	28·0	0·05	0·05-0·11	1·1-9·9
N06AB05	Paroxetine**	Antidepressant	3·0	0·01	0·01-0·05	2·7
N06AB06	Sertraline	Antidepressant	22·0	0·05	0·05-0·25	1·5-97·0
N06AB08	Fluvoxamine**	Antidepressant	13·0	0·02	0·06-0·23	1·7
N06AB10	Escitalopram	Antidepressant	28·0	0·05	0·05-0·11	1·1-9·9
N06AX03	Mianserin	Antidepressant	21·0	0·05	0·015·0·07	
N06AX11	Mirtazapine	Antidepressant	20·0	0·02	0·03-0·50	0·7-5·8
N06AX12	Bupropion**	Antidepressant	20·0	0·02	0·05-0·10	
N07BA02	Bupropion**	Antidepressant	20·0	0·02	0·05-0·10	
N06AX16	Venlaflaxine	Antidepressant	9·0	0·05	0·10-0·40	1·5-5·0
N06AX18	Reboxetine**	Antidepressant	13·0	0·02	0·06-0·35	0·8
N06AX21	Duloxetine**	Antidepressant	8·0	0·03	0·03-0·12	1·5

* Method T1, 2011-09-01--2013-12-31 ** Excluded substances when lack of routine testing throughout study period, or insufficient limit of detection.

^1^ ATC: Anatomical Therapeutic Chemical

^2^ Half-lives as officially reported by drug manufacturers to Läkemedelsindustriföreningen (http://www.fass.se 2017-04-05)

^3^ Detection thresholds (µg/ml = mg/L) for liquid-chromatography time-of-flight mass spectrometry at the National Board of Forensic Medicine’s toxicologicy unit in Linköping, Sweden (Valideringsbeskrivning för metod T01, B-läkemedel och droger. Ledningssystem För Rättsgenetik Och Rättskemi Dokument VT001 2016).

^4^ Schulz M, Iwersen-Bergmann S, Andresen H, Schmoldt A. Therapeutic and toxic blood concentrations of nearly 1,000 drugs and other xenobiotics. Crit Care 2012; 16: 1–4.

^5^ Leikin JB, Watson WA. Post-mortem Toxicology: What The Dead Can And Cannot Tell Us, Journal of Toxicology: Clinical Toxicology. J Toxicol 2003; 41: 47–56.

^6^ McIntyre IM. Liver and peripheral blood concentration ratio (L/P) as a marker of postmortem drug redistribution: A literature review. Forensic Sci Med Pathol 2014; 10: 91–6.

^7^ Zilg B, Thelander G, Giebe B, Druid H. Postmortem blood sampling—Comparison of drug concentrations at different sample sites. Forensic Sci Int 2017; 278: 296–303

## Data Availability

The data supporting the findings of this study are not publicly available due to ethical and legal restrictions but are available from the corresponding author upon reasonable request and subject to approval by the relevant ethics committee and data custodian.

