# Supplementary figures and images for: Adherence to psychotropic medication in completed suicide in Sweden 2006–2013: a forensic-toxicological matched case-control study
Source: Eur J Clin Pharmacol. 2019 Jun 19;75(10):1421–30. doi: 10.1007/s00228-019-02707-z (PMC12913278; doi:10.1007/s00228-019-02707-z)

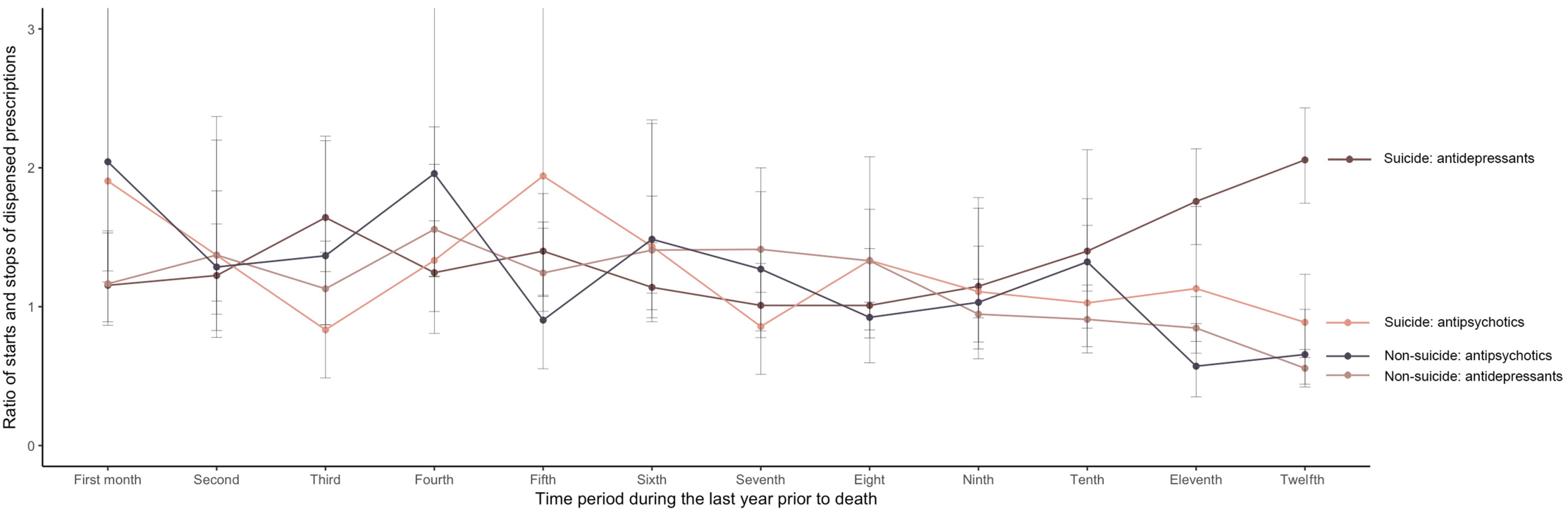

Supplement: Supplementary file 1 — (PDF 2128 kb) [file 228_2019_2707_MOESM1_ESM.pdf]
