# Supplementary material for: Adherence to psychotropic medication in completed suicide in Sweden 2006–2013: a forensic-toxicological matched case-control study
Source: Eur J Clin Pharmacol. 2019 Jun 19;75(10):1421–30. doi: 10.1007/s00228-019-02707-z (PMC12913278; doi:10.1007/s00228-019-02707-z)
Supplement: Supplementary file 2 — (PDF 1015 kb) [file 228_2019_2707_MOESM2_ESM.pdf]

Supplementary Table 1. Main causes of death

Proportions of main causes of death (N)	Non-suicide controls	Completed suicide cases
A01-B99 (Certain infectious and parasitic diseases)	0·00% (0)	0·26% (26)
C00-D48 (Neoplasms)	0·04% (2)	1·30% (122)
D50-D89 (Diseases of the blood and blood-forming organs and certain disorders involving the immune mechanism)	0·00% (0)	0·06% (6)
E00-E90 (Endocrine, nutritional, and metabolic diseases)	0·09% (5)	2·74% (271)
F00-F99 (Mental and behavioral disorders)	0·08% (4)	5·23% (517)
G00-G99 (Diseases of the nervous system)	0·02% (1)	1·98% (196)
H00-H99 (Diseases of the eye and adnexa)	0·00% (0)	0·00% (0)
H60-H95 (Diseases of the ear and mastoid process)	0·00% (0)	0·00% (0)
I00-I99 (Diseases of the circulatory system)	0·83% (44)	37·18% (3673)
J00-J99 (Diseases of the respiratory system)	0·06% (3)	4·40% (435)
K00-K93 (Diseases of the digestive system)	0·19% (10)	5·39% (532)
L00-L99 (Diseases of the skin and subcutaneous tissue)	0·00% (0)	0·02% (2)
M00-M99 (Diseases of the musculoskeletal system and connective tissue)	0·00% (0)	0·02% (2)
N00-N99 (Diseases of the genitourinary system)	0·00% (0)	0·14% (14)
O00-O99 (Pregnancy, childbirth, and the puerperium)	0·00% (0)	0·01% (1)
P00-P99 (Certain conditions originating in the perinatal period)	0·00% (0)	0·00% (0)
Q00-Q99 (Congenital malformations, deformations, and chromosomal abnormalities)	0·00% (0)	0·18% (18)
R00-R99 (Symptoms, signs, and abnormal clinical and laboratory findings, not elsewhere classified)	0·51% (27)	2·45% (242)
S00-T98 (Injury, poisoning, and certain other consequences of external causes)	0·00% (0)	0·00% (0)
V01-Y98 (External causes of morbidity and mortality	98·19% (5198)	38·69% (3822)

Supplementary Table 2. Included ICD-10 codes for comorbid conditions contributing to causes of death and occurring during inpatient care

Diagnosis/comorbid condition	ICD-10
Diabetes mellitus	E10.0-E14.9; E89.1
Alcohol-related disorders	F10.0; K70.0-K70.9; K85.0-K86.9
Drug-related disorders	F19.0-F19.9
Psychosis	F20.0-F29.9
Bipolar disorder	F30.0-F31.9
Depression	F30.0-F31.9
Heart failure	I11.0; I42.0-I43.9; I50.0-I50.9
Hypertension	I10.0-I11.9
Ischemic heart disease	I20.0-I25.9
Asthma/chronic obstructive pulmonary disease	J44.0-J46.9

Supplementary table 3. Elimination half-lives, detection thresholds, therapeutic ranges, and postmortem redistribution factors for the studied drugs

ATC [1]	Substance	ATC class [1]	Minimum elimination half-life (h) [2]	Detection threshold (mg/L) [3]	Therapeutic range (mg/L) [4]	Postmortal redistribution factor [5-7]
N05AA01	Chlorpromazine	Antipsychotic	30·0	0·10	0·03-0·10	4·0
N05AB01	Dixyrazine	Antipsychotic	5·0	0·20	0·30--	
N05AC02	Thioridazine	Antipsychotic	21·0	0·20	0·20-2·00	1·2
N05AD01	Haloperidol	Antipsychotic	12·0	0·01	0·005-0·017	3·6
N05AE04	Ziprasidone	Antipsychotic	6·5	0·01	0·05-0·20	
N05AF05	Zuclopenthixol	Antipsychotic	20·0	0·01	0·004-0·05	
N05AH02	Clozapine	Antipsychotic	6·0	0·05	0·10-0·60	2·8
N05AH03	Olanzapine	Antipsychotic	30·0	0·02	0·02-0·08	2·7-23·0
N05AH04	Quetiapine	Antipsychotic	7·0	0·02	0·10-0·50	9·0
N05AX08	Risperidone	Antipsychotic	23·0	0·01	0·006-0·02	
N05AX12	Aripiprazole	Antipsychotic	25·0	0·02	0·15-0·50	0·4
N05AX13	Paliperidone	Antipsychotic	23·0	0·01	0·02-0·06	
N06AA04	Clomipramine	Antidepressant	21·0	0·05	0·02-0·40	1·9
N06AA09	Amitriptyline	Antidepressant	25·0	0·05	0·05-0·30	
N06AA10	Nortriptyline	Antidepressant	18·0	0·10	0·02-0·20	
N06AA21	Maprotiline	Antidepressant	27·0	0·10	0·075-0·130	3·4
N06AC01	Maprotiline	Antidepressant	27·0	0·10	0·075-0·130	3·4
N06AB03	Fluoxetine	Antidepressant	96·0	0·02	0·12-0·50	1·6-2·9
N06AB04	Citalopram	Antidepressant	28·0	0·05	0·05-0·11	1·1-9·9
N06AB05	Paroxetine	Antidepressant	3·0	0·01	0·01-0·05	2·7
N06AB06	Sertraline	Antidepressant	22·0	0·05	0·05-0·25	1·5-97·0
N06AB08	Fluvoxamine	Antidepressant	13·0	0·02	0·06-0·23	1·7
N06AB10	Escitalopram	Antidepressant	28·0	0·05	0·05-0·11	1·1-9·9
N06AX03	Mianserin	Antidepressant	21·0	0·05	0·015·0·07	
N06AX11	Mirtazapine	Antidepressant	20·0	0·02	0·03-0·50	0·7-5·8
N06AX12	Bupropion	Antidepressant	20·0	0·02	0·05-0·10	
N07BA02	Bupropion	Antidepressant	20·0	0·02	0·05-0·10	
N06AX16	Venlaflaxine	Antidepressant	9·0	0·05	0·10-0·40	1·5-5·0
N06AX18	Reboxetine	Antidepressant	13·0	0·02	0·06-0·35	0·8
N06AX21	Duloxetine	Antidepressant	8·0	0·03	0·03-0·12	1·5

[1] ATC: Anatomical Therapeutic Chemical

[2] Half-lives as officially reported by drug manufacturers to Läkemedelsindustriföreningen (http://www.fass.se 2017-04-05)

[3] Detection thresholds (µg/ml = mg/L) for liquid-chromatography time-of-flight mass spectrometry at the National Board of Forensic Medicine's toxicologicy unit in Linköping, Sweden (Valideringsbeskrivning för metod T01, B-läkemedel och droger. Ledningssystem För Rättsgenetik Och Rättskemi Dokument VT001 2016).

[4] Schulz M, Iwersen-Bergmann S, Andresen H, Schmoldt A. Therapeutic and toxic blood concentrations of nearly 1,000 drugs and other xenobiotics. Crit Care 2012; 16: 1–4.

[5] Leikin JB, Watson WA. Post-mortem Toxicology: What The Dead Can And Cannot Tell Us, Journal of Toxicology: Clinical Toxicology. J Toxicol 2003; 41: 47–56.

[6] McIntyre IM. Liver and peripheral blood concentration ratio (L/P) as a marker of postmortem drug redistribution: A literature review. Forensic Sci Med Pathol 2014; 10: 91–6.

[7] Zilg B, Thelander G, Giebe B, Druid H. Postmortem blood sampling—Comparison of drug concentrations at different sample sites. Forensic Sci Int 2017; 278: 296–303

Supplementary table 4. Changes, dispensation ratios, and dispensed daily defined doses during year prior to death

**All prescriptions**	**Cases – completed suicide**					**Controls – non-suicidal death**				
**Month, year preceding death**	**Changes /person**	**Ratio**	**95% CI**	**Change in ratio from previous month**	**DDD /person**	**Changes /person**	**Ratio**	**95% CI**	**Change in ratio from previous month**	**DDD /person**
First	0·56	1·15	1·07-1·24	NA	194	1·03	1·15	1·09-1·21	NA	242
Second	0·57	1·12	1·04-1·20	−3%	200	1·03	1·13	1·07-1·20	−2%	251
Third	0·56	1·19	1·11-1·28	6%	214	1·08	1·13	1·08-1·20	0%	259
Fourth	0·57	1·21	1·13-1·30	2%	210	1·08	1·22	1·16-1·28	8%	262
Fifth	0·62	1·23	1·15-1·32	2%	220	1·09	1·12	1·06-1·18	−8%	259
Sixth	0·66	1·21	1·13-1·29	−2%	235	1·14	1·19	1·13-1·25	6%	278
Seventh	0·65	1·05	0·98-1·12	−13%	238	1·17	1·14	1·08-1·20	−4%	282
Eight	0·65	1·05	0·98-1·12	0%	245	1·17	1·02	0·97-1·07	−11%	284
Ninth	0·70	1·08	1·01-1·15	3%	254	1·17	0·96	0·91-1·00	−6%	284
Tenth	0·71	1·08	1·01-1·15	0%	263	1·22	0·93	0·89-0·98	−3%	299
Eleventh	0·84	1·06	1·00-1·12	−2%	269	1·31	0·80	0·76-0·84	−14%	305
Twelfth	1·10	0·96	0·92-1·01	−9%	285	1·49	0·66	0·64-0·70	−18%	299
Death	-	-	-	-	-	-	-	-	-	-
Changes of ratios (6 months)	Slope: −0·0120; SD: 0·0103; p=0·3084					Slope: −0·0883; SD: 0·009; p=0·0006				
Changes of ratios (12 months)	Slope: −0·0166; SD: 0·0050; p=0·0083					Slope: −0·0399; SD: 0·0081; p=0·0006				

All psychotropic medications	Cases – completed suicide					Controls – non-suicidal death				
Month, year preceding death	Changes /person	Ratio	CI	Change in ratio from previous month	DDD /person	Changes /person	Ratio	CI	Change in ratio from previous month	DDD /person
First	0·18	1·17	1·04-1·35	NA	73	0·27	1·11	0·98-1·22	NA	84
Second	0·18	1·12	1·00-1·29	−4%	77	0·27	1·25	1·14-1·41	13%	84
Third	0·18	1·33	1·17-1·52	19%	79	0·27	1·14	0·99-1·23	−9%	89
Fourth	0·19	1·12	1·00-1·29	−16%	79	0·28	1·30	1·17-1·44	14%	89
Fifth	0·20	1·26	1·13-1·44	13%	82	0·27	1·17	1·04-1·28	−10%	89
Sixth	0·21	1·19	1·06-1·35	−6%	87	0·30	1·30	1·19-1·45	11%	95
Seventh	0·12	1·06	0·93-1·18	−11%	92	0·31	1·14	1·04-1·27	−12%	95
Eight	0·20	1·17	1·03-1·31	10%	92	0·30	1·10	0·98-1·19	−4%	97
Ninth	0·21	1·23	1·11-1·40	5%	95	0·29	0·98	0·87-1·07	−11%	100
Tenth	0·25	1·32	1·17-1·46	7%	97	0·30	0·99	0·90-1·10	1%	102
Eleventh	0·32	1·48	1·35-1·65	12%	102	0·31	0·82	0·74-0·90	−17%	103
Twelfth	0·48	1·42	1·30-1·52	−4%	110	0·39	0·67	0·61-0·73	−18%	104
Death	-	-	-	-	-	-	-	-	-	-
Changes of ratios (6 months)	Slope: 0·08057; SD: 0·0123; p=0·0028					Slope: −0·09086; SD: 0·01306; p=0·0022				
Changes of ratios (12 months)	Slope: 0·02122; SD: 0·0089; p=0·0389					Slope: −0·04101; SD: 0·01043; p=0·0028				

Toxicologically verifiable psychotropic medications	Cases – completed suicide					Controls – non-suicidal death				
Month, year preceding death	Changes /person	Ratio	CI	Change in ratio from previous month	DDD /person	Changes /person	Ratio	CI	Change in ratio from previous month	DDD /person
First	0·05	1·30	1·00-1·64	NA	34	0·05	1·29	1·03-1·65	NA	34
Second	0·05	1·21	1·04-1·68	−7%	37	0·05	1·35	1·09-1·72	5%	34
Third	0·05	1·43	1·15-1·89	18%	38	0·06	1·18	0·91-1·41	−13%	35
Fourth	0·05	1·27	1·01-1·62	−11%	38	0·06	1·63	1·26-1·97	38%	35
Fifth	0·05	1·44	1·20-1·96	13%	39	0·06	1·16	0·90-1·39	−29%	36
Sixth	0·06	1·18	0·93-1·46	−18%	40	0·07	1·43	1·12-1·70	23%	39
Seventh	0·05	0·95	0·79-1·27	−19%	42	0·07	1·38	1·06-1·61	−3%	39
Eight	0·06	1·10	0·87-1·39	16%	43	0·06	1·21	0·96-1·49	−12%	41
Ninth	0·07	1·15	0·95-1·42	5%	44	0·07	0·96	0·73-1·10	−21%	42
Tenth	0·07	1·30	1·04-1·60	13%	45	0·06	0·99	0·79-1·21	3%	42
Eleventh	0·10	1·62	1·34-1·92	25%	46	0·07	0·78	0·63-0·95	−21%	44
Twelfth	0·15	1·76	1·51-2·04	9%	51	0·08	0·59	0·48-0·70	−24%	44
Death	-	-	-	-	-	-	-	-	-	-
Changes of ratios (6 months)	Slope: 0·1646; SD: 0·01850; p=0·0009					Slope: −0·1489; SD: 0·01585; p=0·0007				
Changes of ratios (12 months)	Slope: 0·02094; SD: 0·01861; p=0·2867					Slope: −0·06087; SD: 0·01686; p=0·0048				

Abbreviations: CI = confidence interval, DDD = Defined Daily Dose

Supplementary table 5. Ratios of initiated and discontinued dispensed prescriptions during the year preceding death

	Completed-suicide cases		Non-suicide controls	
**Antidepressants**				
**Month**	**Ratio**	**CI**	**Ratio**	**CI**
First	1·15	0·87-1·55	1·16	0·89-1·53
Second	1·22	0·95-1·60	1·37	1·04-1·83
Third	1·64	1·25-2·19	1·13	0·87-1·47
Fourth	1·24	0·96-1·62	1·56	1·21-2·02
Fifth	1·40	1·07-1·81	1·24	0·97-1·61
Sixth	1·14	0·89-1·46	1·41	1·10-1·80
Seventh	1·01	0·78-1·31	1·41	1·10-1·83
Eight	1·01	0·78-1·31	1·33	1·03-1·70
Ninth	1·15	0·92-1·44	0·95	0·75-1·20
Tenth	1·40	1·11-1·78	0·91	0·71-1·16
Eleventh	1·76	1·45-2·14	0·85	0·66-1·07
Twelfth	2·06	1·74-2·43	0·56	0·44-0·69
**Antipsychotics**				
**Month**	**Ratio**	**CI**	**Ratio**	**CI**
First	1·90	1·18-3·36	2·04	1·26-3·67
Second	1·37	0·83-2·37	1·29	0·78-2·20
Third	0·83	0·49-1·39	1·37	0·87-2·23
Fourth	1·33	0·81-2·29	1·96	1·22-3·44
Fifth	1·94	1·08-3·55	0·90	0·55-1·57
Sixth	1·43	0·92-2·32	1·49	0·98-2·35
Seventh	0·86	0·51-1·41	1·27	0·83-2·00
Eight	1·33	0·83-2·08	0·92	0·60-1·42
Ninth	1·11	0·70-1·79	1·03	0·62-1·71
Tenth	1·03	0·67-1·59	1·32	0·85-2·13
Eleventh	1·13	0·75-1·72	0·57	0·35-0·88
Twelfth	0·89	0·63-1·23	0·66	0·42-0·98
